# A Multicenter Study on Expressions of Vascular Endothelial Growth Factor, Matrix Metallopeptidase-9 and Tissue Inhibitor of Metalloproteinase-2 in Oral and Maxillofacial Squamous Cell Carcinoma

**DOI:** 10.5812/ircmj.13185

**Published:** 2014-03-05

**Authors:** Min Li, Zhiying Wang, Yang Xing, Jin Yu, Luming Tian, Dianming Zhang, Zengxi Xin

**Affiliations:** 1 Department of Oral Implantology, Second Affiliated Hospital of Liaoning Medical University, Jinzhou, China; 2 Jinzhou City Oral Cavity Hospital, Jinzhou, China; 3 Department of Prosthodontics, Second Affiliated Hospital of Liaoning Medical University, Jinzhou, China; 4 Department of Oral and Maxillofacial Surgery, Second Affiliated Hospital of Liaoning Medical University, Jinzhou, China; 5 Department of Stomatology, Hospital of Liaoning University of Technology, Jinzhou, China

**Keywords:** Oral Surgical Procedures, Carcinoma, Squamous Cell, Vascular Endothelial Growth Factor A, Matrix Metalloproteinase 9, Tissue Inhibitor of Metalloproteinase-2

## Abstract

**Background:**

Vascular endothelial growth factor (VEGF), matrix metallopeptidase-9 (MMP-9) and tissue inhibitor of metalloproteinase-2 (TIMP-2) are potential markers of oral and maxillofacial squamous cell carcinoma (SCC).

**Objectives:**

To explore the association between expression of VEGF, MMP-9 and TIMP-2 in oral and maxillofacial SCC and clinicopathological factors.

**Patients and Methods:**

Immunohistochemical Envision method was used to analyze the expression of VEGF, MMP-9 and TIMP-2 in 54 cases of oral and maxillofacial SCC and the association with clinicopathological factors such as clinical staging and lymphatic metastasis.

**Results:**

Brownish-yellow staining is correlated with positive expression of VEGF, MMP-9 and TIMP-2. Positive expression of VEGF and MMP-9 was correlated with lymphatic metastasis, and their positive expression rates were significantly higher in patients with lymphatic metastasis than those without it (VEGF: χ^2^ = 30.00; P = 0.001; MMP-9: χ^2^ = 18.27, P = 0.001). The positive expression rate of MMP-9 decreased at earlier clinical stages (P < 0.05). Positive expression of TIMP-2 was correlated with lymphatic metastasis, clinical staging and T classification. The positive rate of TIMP-2 expression in patients with lymphatic metastasis was significantly lower than those without it (χ^2^ = 26.74, P = 0.002), which significantly reduced with increasing clinical stage and T classification (P < 0.05).

**Conclusions:**

Lymphatic metastasis in patients with oral and maxillofacial SCC is closely related to the positive expression of VEGF, MMP-9 and TIMP-2. MMP-9 and TIMP-2 can affect the progression of cancer, which is valuable for studies on oral and maxillofacial SCC genes.

## 1. Background

Maxillofacial malignant tumors are mainly carcinomas instead of sarcomas in China. Among carcinomas, the incidence rate of squamous cell carcinoma (SCC) ranks first at about 80% (1, 2). Currently, oral and maxillofacial SCC is mainly treated by surgery and radiotherapy, but the effect is unsatisfactory, thus demanding targeted therapy. Tumor stem cells in oral and maxillofacial SCC, such as vascular endothelial growth factor (VEGF), phosphatase and tensin homolog (PTEN), matrix metallopeptidase-9 (MMP-9), tissue inhibitor of metalloproteinase-2 (TIMP-2), CD44 and CD133, have been potential markers (3) by exerting corresponding effects on the occurrence and suppression of tumors or lymphatic metastasis. Tumor markers (4), produced by tumors themselves, include embryonic antigen, carbohydrate antigen, related enzymes, hormones and gene, and some others. The content changes of the markers indicate the properties of tumors, providing references for further treatment by preliminarily suggesting the changes of tumor tissues as well as the differentiation and function of cells. For instance, the markers for lung cancer are CEA, CYFRA21-1 and NSE, and those for prostate cancer are f-PSA and t-PSA. However, the markers often function through the synergetic effects of molecules such as CD44 and CD133, PTEN and MMP-9, and some others, contributing to the advent of "gene synergistic hypothesis".

## 2. Objectives

This study aimed to investigate the expression levels of VEGF, MMP-9 and TIMP-2 in 54 patients with oral and maxillofacial SCC in our medical institutions based on gene synergistic hypothesis to explore its association with clinicopathological factors.

## 3. Patients and Methods

### 3.1. Clinical Information

The human research ethics committees of our institutions approved the study, and an informed consent was obtained from all participants. Sixty patients with maxillofacial SCC were treated from April 2011 to April 2013, of which 54 cases with primary foci and without metastases to other organs were included in this retrospective clinical study, consisting of 40 males and 14 females aged 26 - 65 years old with an average age of 50.3. All the included patients had complete records of clinical data. There were 6 cases of tongue cancer, 6 cases of floor-of-mouth cancer, 33 cases of cheek cancer and 10 cases of gum cancer. According to the TNM staging criteria for oral squamous cell carcinoma, there were 26 cases of T1-T2 stage and 28 cases of T3-T4 stage. According to the clinical staging criteria established by UICC, there were 18 cases of I-II stage and 36 cases of III-IV stage.

### 3.2. Sampling Strategy

#### 3.2.1. Sampling Center

The observer of this study, Dr. Lili Wang, who is not one of the coauthors, is actually affiliated to Second Affiliated Hospital of Liaoning Medical University (Jinzhou, China) and PLA General Hospital (Beijing, China). Therefore, the clinical data in this study was collected from two locations (Jinzhou and Beijing) and the pathological departments of more than three medical institutions.

#### 3.2.2. Sampling Guideline

Primary maxillofacial SCC; without metastases; complete clinical data; informed consent was obtained from all enrolled research participants

#### 3.2.3. Sampling Methods

The mucosae of the 54 cases with lesions were sampled, fixed in 10% formaldehyde, embedded with paraffin, and prepared into sections.

### 3.3. Immunohistochemical Envision Two-Step Staining

#### 3.3.1. Main Materials

Envision Flex (+) detection kit and ready-to-use (RTU) primary antibody reagent used in Envision method were purchased from Beijing XiYa JinQiao Biological Technology Co., Ltd.; horseradish catalase DAB chromogenic reagent kit was obtained from Tianjin Baihao Biological Technology Co., Ltd.; polylysine solution was obtained from Fuzhou Maixin Biological Technology Development Co., Ltd.

VEGF and MMP-9 mouse anti-human monoclonal antibodies were provided by Shanghai Huayi Biological Technology Co., Ltd. TIMP-2 rabbit anti-human polyclonal antibody was obtained from Beijing Zhongshan Golden Bridge Biotechnology Co., Ltd.

#### 3.3.2. Two-Step Staining Method

Paraffin sections of cancer cells were placed in a 67°C incubator for deparaffinage, taken out after about 2h, and then washed with PBS (pH 7.4) three times (3 min/time) (4-7). The sections were placed onto plastic shelves, placed into microwave-boiling citrate buffer (pH 6.0), taken out after boiling by medium wave for about 10 min, cooled by flowing water, and washed with PBS twice (3 min/time). Each section was drop wise added H_2_O_2_, and washed with PBS three times at room temperature after 10 min (3 min/time). Then PBS was removed, and each section was added primary antibody and incubated at room temperature, and washed with PBS five times after 2h (3 min/time). PBS was removed again, and each section was added avidin, incubated for 20 min, and then washed with PBS. After PBS was removed, each section was added polymer enhancer and washed after 20 min. After removal of PBS, each section was added appropriate anti-mouse/rabbit polymer, incubated for 30 min and washed with PBS. After PBS was removed again, and each section was added DAB and observed by microscope. After hematoxylin counter staining, HCl differentiation, bluing after tap water washing, drying and clearing, sections were sealed with vegetable gum and observed after being dried in air.

### 3.4. Observation Indices

#### 3.4.1. Positive Expression

VEGF, MMP-9 and TIMP-2 were regarded as positive expressions in oral and maxillofacial SCC when sections showed brownish yellow after staining. Yellow particulate matters appeared in the cytoplasm (8) could be observed by microscope. At the same time, primary antibody was replaced with PBS as the negative control. Five cell visual fields were selected, with about 200 cells in each field on average to calculate the percentage of positive tumor cells to total ones. The percentage of positive cells equal to or higher than 10% was considered positive (+), and the opposite indicated negative (-) (9).

#### 3.4.2. Clinicopathological Factors

Preoperative and postoperative cervical lymph node dissection samples were observed after HE staining (10), and sent to pathology laboratory for expert assistance to determine the occurrence of lymphatic metastasis. Clinical records of patients were reviewed to analyze the association between the positive expressions of VEGF, MMP-9 and TIMP-2 in oral and maxillofacial SCC and clinical staging and T classification. 

### 3.5. Statistical Analysis

All data was analyzed by SPSS 17.0 and subjected to Chi Square test. P < 0.05 was considered statistically significant.

## 4. Results

### 4.1. VEGF Expression

**Figure 1.**
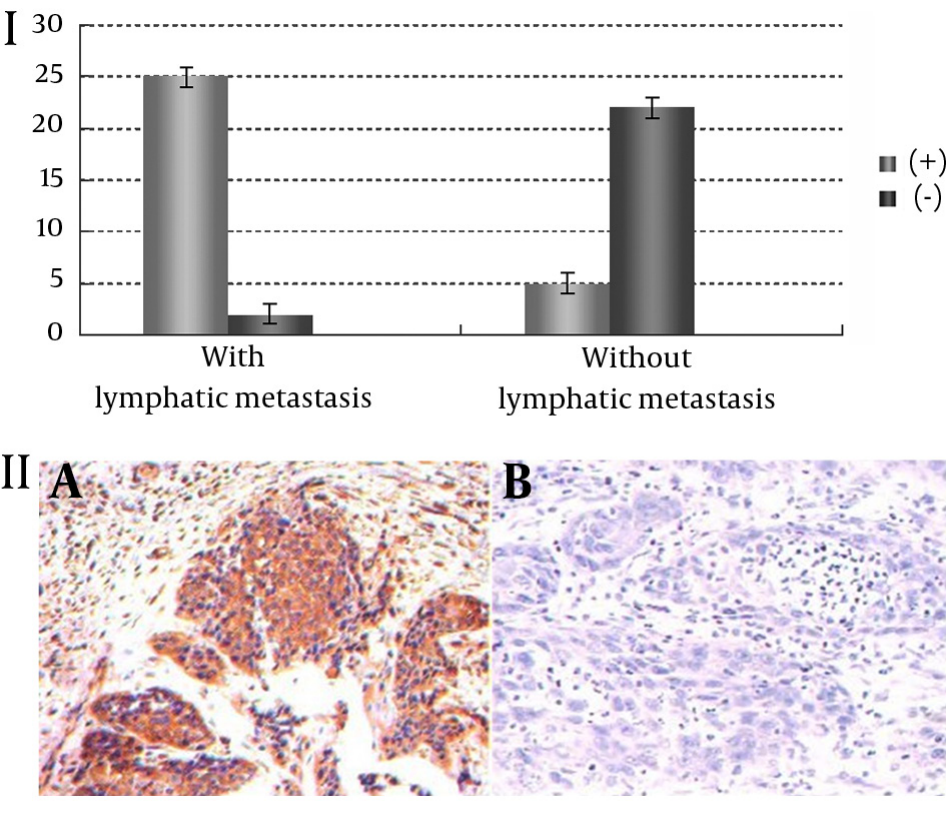
I: Association between VEGF positive expression and lymphatic metastasis; II: expression of VEGF in lymph nodes. A: With lymphatic metastasis, positive expression (+); B: Without lymphatic metastasis, negative expression (-).

Positive expression of VEGF was associated with lymphatic metastasis, and the positive rate of VEGF expression was significantly higher in patients with lymphatic metastasis than those without it (χ^2^ = 30.00, P = 0.001). Positive VEGF expression had no association with gender, age, cancerous part, clinical staging and T classification (P > 0.05) (Table 1, Figure 1). 

**Table 1.** Analysis of VEGF Positive Expression and Related Factors ^a^

Related factor	n	Positive (+)	Negative (-)	χ^2^	P Value
**Gender**					0.832 ^b^
Male	40	22 (55.0)	18 (45.0)	0.02	
Female	14	8 (57.1)	6 (42.9)		
**Age, y**					0.376 ^b^
26 - 50	25	14 (56.0)	11 (44.0)	0.00	
50 - 65	29	16 (55.2)	13 (44.8)		
**Cancerous part**					0.921 ^b^
Tongue cancer	6	2 (33.3)	4 (66.7)	0.01	
Floor-of-mouth cancer	6	3 (50.0)	3 (50.0)		
Cheek cancer	33	19 (57.6)	14 (42.4)		
Gum cancer	10	6 (60.0)	4 (40.0)		
**Clinical stage**					> 0.999 ^b^
I - II	18	10 (55.6)	8 (44.4)	0.00	
III - IV	36	20 (55.6)	16 (44.4)		
**T classification**					0.982 ^b^
T1 - T2	26	15 (57.7)	11 (42.3)	0.09	
T3 - T4	28	15 (53.6)	13 (46.4)		
**Lymphatic metastasis**					0.001 ^c^
Yes	27	25 (92.6)	2 (7.4)	30.00	
No	27	5 (18.5)	22 (81.5)		

^a^ Data are presented as No. (%).

^b^ Without significant differences.

^c^ With Significant Difference.

### 4.2. MMP-9 Expression

**Figure 2.**
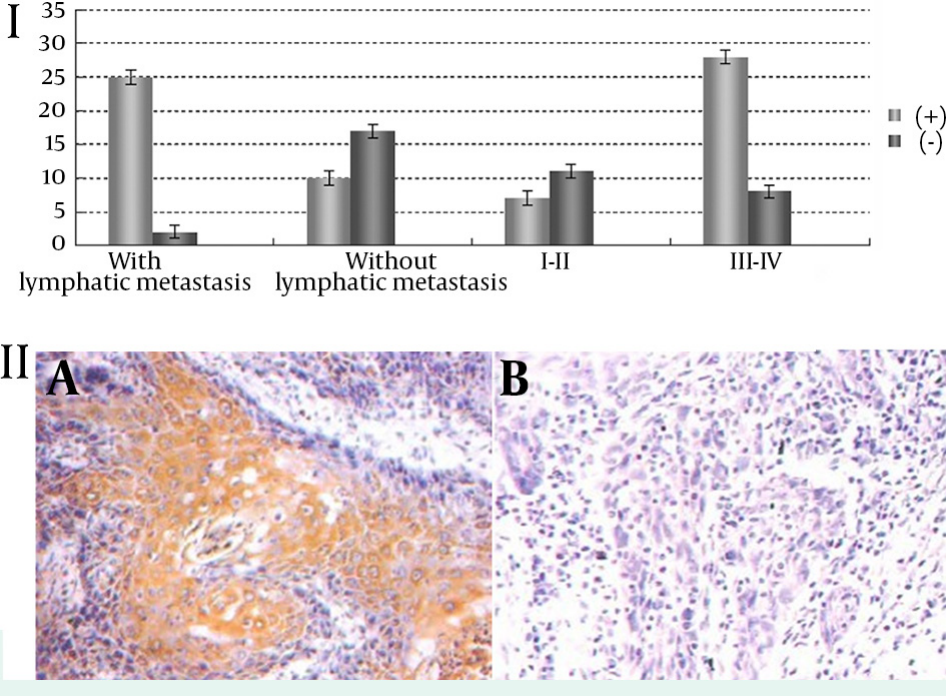
I: Association between MMP-9 positive expression and lymphatic metastasis and clinical stages; II: expression of MMP-9 in lymph nodes. A: With lymphatic metastasis, positive expression (+); B: Without lymphatic metastasis, negative expression (-).

Positive expression of MMP-9 was related with lymphatic metastasis, and the positive rate of MMP-9 expression was significantly higher in patients with lymphatic metastasis than those without it (χ^2^ = 18.27, P = 0.001). Meanwhile, the positive expression rate of MMP-9 increased with increasing clinical staging (χ^2^ = 7.96, P = 0.028). Its positive expression had no association with gender, age and other factors (P > 0.05) (Table 2, Figure 2). 

**Table 2.** Analysis of MMP-9 Positive Expression and Related Factors ^a^

Related Factor	n	Positive (+)	Negative (-)	χ^2^	P Value
**Gender**					0.737 ^b^
Male	40	25 (62.5)	15 (37.5)	0.36	
Female	14	10 (71.4)	4 (28.6)		
**Age**					0.821 ^b^
26-50	25	17 (68.0)	8 (32.0)	0.21	
50-65	29	18 (62.1)	11 (37.9)		
**Cancerous part**					0.691 ^b^
Tongue cancer	6	5 (83.3)	1 (16.7)	0.26	
Floor-of-mouth cancer	6	4 (66.7)	2 (33.3)		
Cheek cancer	33	20 (60.6)	13 (39.4)		
Gum cancer	10	6 (60.0)	4 (40.0)		
**Clinical stage**					0.028 ^b^
I - II	18	7 (38.9)	11 (61.1)	7.96	
III - IV	36	28 (77.8)	8 (22.2)		
**T classification**					0.882 ^b^
T1 - T2	26	17 (65.4)	9 (34.6)	0.01	
T3 - T4	28	18 (64.3)	10 (35.6)		
**Lymphatic metastasis**					0.001 ^c^
Yes	27	25 (92.6)	2 (7.4)	18.27	
No	27	10 (37.0)	17 (63.0)		

^a^ Data are presented as No. (%).

^b^ Without significant differences.

^c^ With significant differences.

### 4.3. TIMP-2 Expression

**Figure 3.**
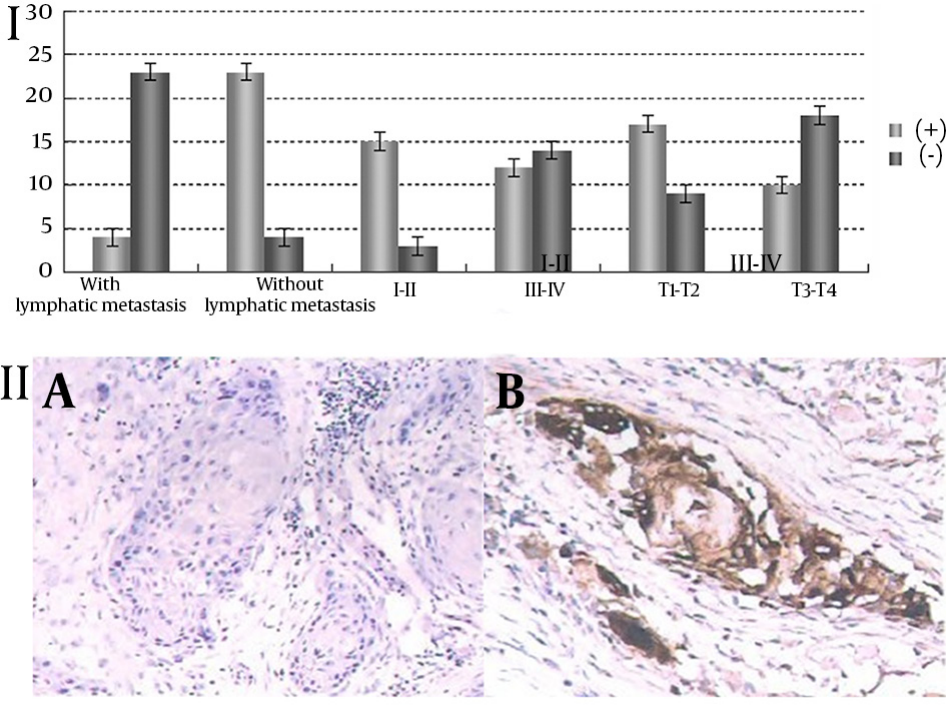
I: Association between TIMP-2 expression and lymphatic metastasis, clinical stages and T classification; II: Expression of TIMP-2 in lymph nodes. A: With lymphatic metastasis, negative expression (-); B: Without lymphatic metastasis, positive expression (+).

Positive expression of TIMP-2 was associated with lymphatic metastasis, clinical staging and T classification. The positive rate of TIMP-2 expression in patients with lymphatic metastasis was significantly lower than those without it (χ^2^ = 26.74, P = 0.002), and the rate significantly reduced with increasing clinical stages and T classification (P < 0.05). (Table 3, Figure 3). 

**Table 3.** Analysis of TIMP-2 Positive Expression and Related Factors

Related factor	n	Positive (+)	Negative (-)	χ^2^	P Value
**Gender**					> 0.999 ^a^
Male	40	20 (50.0)	20 (50.0)	0.00	
Female	14	7 (50.0)	7 (50.0)		
**Age**					0.902 ^a^
26-50	25	12 (48.0)	13 (52.0)	0.07	
50-65	29	15 (51.7)	14 (48.3)		
**Cancerous part**					0.935 ^a^
Tongue cancer	6	5 (83.3)	1 (16.7)	0.23	
Floor-of-mouth cancer	6	2 (33.3)	4 (66.7)		
Cheek cancer	33	24 (72.7)	9 (27.3)		
Gum cancer	10	7 (70.0)	3 (30.0)		
**Clinical stage**					0.028 ^a^
I - II	18	15 (83.3)	3 (16.7)	12.00	
III - IV	36	12 (33.3)	14 (66.7)		
**T classification**					0.048 ^a^
T1 - T2	26	17 (65.4)	9 (34.6)	4.75	
T3 - T4	28	10 (35.7)	18 (64.3)		
**Lymphatic metastasis**					0.002 ^b^
Yes	27	4 (14.8)	23 (85.2)	26.74	
No	27	23 (85.2)	4 (14.8)		

^a^ Without significant differences.

^b^ with significant differences.

## 5. Discussion

We found that VEGF, MMP-9 and TIMP-2 expression was associated with the clinical diagnosis, treatment and prevention of maxillofacial SCC. This study comprehensively revealed the effects of age, gender, lymphatic metastasis, clinical staging and T classification on the expression of VEGF, MMP-9 and TIMP-2. Meanwhile, as a retrospective study, the sample size was not sufficiently large, but the data was collected from more than three medical institutions. Therefore, we believe that the results are representative. Moreover, only the cases with primary foci and without metastases to other organs were included in this retrospective clinical study to assure its accuracy. However, a complicated staining method was used, which may involve operational errors because mucosae were sampled only once and did not allow a second staining. Regardless, the results herein are eligible and valid because the operational errors ranged between (x ± 1).

VEGF, highly expressed in cancer cells, particularly in liver ones, essentially regulates the development of blood vessels, while promoting the migration of mononuclear macrophages (11, 12). Therefore, VEGF has relatively high positive expression in cancer cells with lymphatic metastasis.

MMP-9 is capable of regulating the release of VEGF (13), so VEGF is also highly expressed in cancer cells with high expression of MMP-9, both of which are related with lymphatic metastasis. Its functions mainly depend on the effects of VEGF on vascular and lymphatic development and its involvement in phagocyte movement.

However, TIMP-2 can inhibit the high expression of MMPs (14), and render the latter unable to regulate the release of VEGF (15), thereby reducing the odds of lymphatic metastasis. In general, VEGF can directly regulate the development of blood and lymph vessels as well as phagocytes to induce lymphatic metastasis. MMP-9 can indirectly regulate VEGF to further induce lymphatic metastasis, while TIMP-2 can inhibit the expressions of MMP-9 and VEGF by inhibiting MMPs. Lymphatic metastasis may finally occur in case of disequilibrium between the three. Therefore, the hypothesis of "genetic synergy" has been widely accepted.

In this study, brownish-yellow staining was correlated with positive expression of VEGF, MMP-9 and TIMP-2. Positive expression of VEGF and MMP-9 was correlated with lymphatic metastasis, the rates of which were both significantly higher in patients with lymphatic metastasis than those without it. In the meantime, the positive expression rate of MMP-9 reduced at earlier clinical stages. Moreover, positive expression of TIMP-2 had correlation with lymphatic metastasis, clinical staging and T classification, the rate of which in patients with lymphatic metastasis was significantly lower than those without it, and significantly decreased with increasing clinical stage and T classification. 

In summary, pathogenesis studies concerning oral and maxillofacial SCC on the genetic level focus on the expressions of relevant genes to discover the mechanism of lymphatic metastasis, i.e. it is closely related to the expression of VEGF, MMP-9 and TIMP-2. Meanwhile, MMP-9 and TIMP-2 could also affect the progression of cancer. Similarly, patients can also be treated based on the study of gene expression being the cutting edge of medical field, which necessitates more investigations.
